# Advances in using biomaterials for repairing thin endometrium

**DOI:** 10.3389/fbioe.2025.1697669

**Published:** 2025-11-18

**Authors:** Huizhen Li, Feihong Hu, Fuchen Xie, Xuedong Chen, Honglian Wu

**Affiliations:** Reproductive Medicine Center, Lishui Hospital of Wenzhou Medical University, The First Affiliated Hospital of Lishui University, Lishui People’s Hospital, Lishui, Zhejiang, China

**Keywords:** biomaterials, thin endometrium, tissue engineering, regenerative medicine, repair

## Abstract

Thin endometrium is one of the main factors leading to infertility and miscarriage. The development of biomaterial technology and its clinical applications have shown good effects in promoting endometrial regeneration, improving blood flow, and enhancing cell adhesion, offering new hope for boosting fertility in patients. Therefore, this article aims to review the pathological mechanisms of thin endometrium, existing treatment methods, and research progress of biomaterials in this field, analyze the effects of different types of biomaterials on thin endometrium, and explore their potential and challenges in clinical applications, providing references for future research directions.

## 1 Introduction

About 15%–25% of infertile women have thin endometrium, and it can be as high as 30% in patients with repeated implantation failure. The incidence in Asia (18.7%) is slightly higher than that in Europe and America (15.2%) (Pabuccu et al., 2022; Zhang Y. et al., 2023), where thin endometrium (TE) is one of the main reasons for repeated implantation failure, usually referring to an endometrial thickness of <7 mm during the mid-menstrual cycle (Wang Y. et al., 2024; Cakiroglu et al., 2023). Thin endometrium not only affects the embryo implantation ability but also significantly impacts clinical pregnancy rates and various adverse pregnancy outcomes. Literature shows that women with EMT <7 mm see their live birth rates drop by around 30%–50% (Ahn et al., 2022; Ganer Herman et al., 2022). Therefore, in-depth exploration of the definition, etiology, and clinical significance of thin endometrium is of great academic and clinical value for improving treatment outcomes in infertility patients.

**FIGURE 1:**
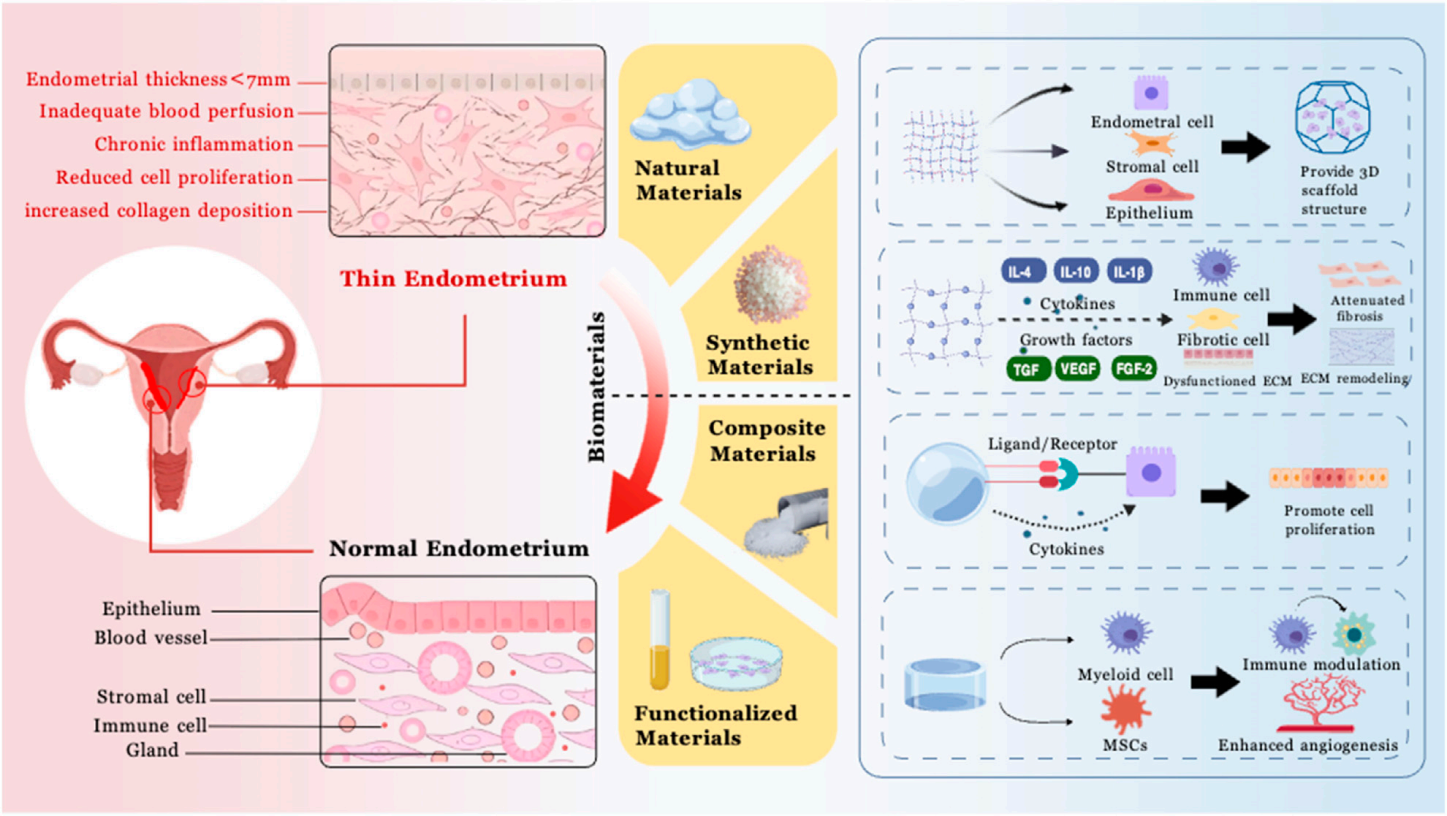
Mechanism diagram: biomaterials in the repair of thin endometrium.

Traditional treatment methods such as high-dose estrogen, vasoactive drugs, and intrauterine infusion have certain effects, but there are issues such as significant individual differences, unstable efficacy, or side effects. In recent years, biomaterials-based regenerative medicine therapies have opened new avenues for the treatment of thin endometrium. Biomaterials, especially strategies combined with mesenchymal stem cells or decellularized scaffolds, have shown great potential in promoting structural repair and functional regeneration of the endometrium in preclinical and clinical studies (Zhang S. et al., 2023; Shi et al., 2023). These materials can mimic the *in vivo* microenvironment, providing physical support for cell attachment, proliferation, and differentiation, and enabling controlled release of growth factors. In the future, with the deep integration of materials science, bioengineering, and clinical medicine, more innovative treatment options are expected to emerge, providing strong support for improving reproductive outcomes in patients with female infertility (Figure 1).

## 2 Pathological mechanisms of thin endometrium

The physiological mechanisms of endometrial dysplasia mainly involve the decreased proliferation ability of endometrial cells and abnormalities in cell cycle signaling pathways. Studies show that single-cell RNA sequencing indicates that the cell cycle signaling pathways in the stromal cells of thin endometrium are inhibited, leading to cellular senescence and excessive collagen deposition, thereby exacerbating the weakness of the endometrium (Lv et al., 2022). However, the causes of this condition are complex, including abnormal hormone levels, inflammatory responses in the endometrium, endocrine disorders, and long-term use of contraceptives (Wang Y. et al., 2024). Additionally, factors such as inflammation and adhesion in the uterine cavity can negatively affect the thickness of the endometrium (Zhang S. et al., 2023).

### 2.1 The impact of hormone levels on endometrial thickness

Studies show that hormone level changes can directly impact how the endometrium grows (Cakiroglu et al., 2023), particularly estrogen and progesterone, which have significant impacts on endometrial thickness. Endometrial cells in patients with thin endometrium also show significant differences in their responsiveness to estrogen, often accompanied by decreased estrogen levels, which directly affects endometrial development, leading to adverse pregnancy outcomes (Wang et al., 2024). Therefore, during *in vitro* fertilization (IVF), the preparation process of the endometrium requires appropriate hormonal support to ensure that the endometrium reaches sufficient thickness to support embryo implantation. Insufficient hormone levels or abnormal hormone secretion can prevent normal endometrial development or reduce endometrial responsiveness, affecting endometrial thickness and function, leading to embryo implantation failure (Chen et al., 2024; Zhu et al., 2022). Thus, changes in hormone levels are important factors affecting thin endometrium.

### 2.2 The role of inflammation and other pathological factors

Inflammatory responses play an important role in the pathological mechanisms of thin endometrium. Chronic inflammatory states can lead to damage and apoptosis of endometrial cells, thereby affecting endometrial thickness and function. Studies have pointed out that in patients with thin endometrium, the levels of inflammatory factors such as IL-1 and IL-6 are significantly elevated, which is closely related to endometrial dysfunction (Lv et al., 2022). Furthermore, the microenvironment of the endometrium is also influenced by the inflammatory status of surrounding tissues; for example, pathological conditions in the uterine cavity such as endometriosis, endometrial adhesions, and endometritis can further exacerbate endometrial dysplasia (Karabay and Ekiz-Yilmaz, 2023). Therefore, inflammation and other pathological factors significantly influence the pathogenesis of thin endometrium by altering the microenvironment and cellular state of the endometrium.

## 3 Treatment options for thin endometrium

### 3.1 Pharmacological treatment

Thin endometrium is an important factor affecting fertility, and medications are key in boosting endometrial thickness and raising pregnancy rates. Studies have shown that granulocyte colony-stimulating factor (G-CSF) and estrogen (E) are common drugs used to treat thin endometrium. G-CSF is believed to improve the condition of thin endometrium by promoting endometrial proliferation and angiogenesis. In a study on rats, G-CSF significantly increased the thickness of the uterine wall and endometrium, demonstrating its significant therapeutic effect on thin endometrium (Kahyaoglu et al., 2024). Additionally, the use of autologous platelet-rich plasma (PRP) for intrauterine injection has also shown good effects, effectively increasing endometrial thickness and improving pregnancy rates (Nazari et al., 2019). In a systematic review, sildenafil citrate was also found to effectively increase endometrial thickness and pregnancy rates in patients with thin endometrium, further supporting the importance of pharmacological treatment in the management of thin endometrium (Li X et al., 2020). However, the efficacy of these drugs varies due to individual differences and is often accompanied by side effects and issues of drug resistance. Many drugs may cause discomfort or more severe health problems while improving endometrial thickness. For example, the use of hormonal drugs may lead to weight gain, mood swings, and other side effects (Park et al., 2021). At the same time, as treatment progresses, some patients may develop drug resistance, leading to decreased treatment effectiveness.

### 3.2 Surgical treatment

Surgical treatment also occupies a certain position in the treatment of thin endometrium, especially in cases where other treatment methods are ineffective. Hysteroscopic surgery plays an important role in treating intrauterine adhesions and repairing endometrial defects, significantly enhancing the safety and effectiveness of the surgery (Fan et al., 2025; Zhang et al., 2022). Hysteroscopic transcervical resection of adhesions (TCRA) has become the standard method for treating severe intrauterine adhesions (IUA), effectively restoring the uterine cavity morphology and partially repairing endometrial damage, creating favorable conditions for endometrial regeneration (Li et al., 2022; Fan et al., 2025). However, although hysteroscopic surgery can effectively remove adhesions after endometrial damage, the repair of thin endometrium is more complex, involving multiple factors such as the regenerative capacity of the endometrium, angiogenesis, and the local hormonal environment. Some studies indicate that thin endometrium may be related to a history of multiple uterine surgeries, and the surgery itself may cause further damage to the endometrium, forming a vicious cycle that affects endometrial regeneration (Zhang S. et al., 2023; Lin et al., 2023). In addition, fibrosis and insufficient blood supply of the endometrium after hysteroscopic surgery also limit the recovery of endometrial thickness, leading to endometrial dysfunction. Single surgical treatment often fails to meet clinical needs. This indicates that exploring new treatment options is urgent, which has also promoted the development of a series of new technologies. Laser surgery utilizes high-energy laser beams for precise cutting, effectively removing diseased tissue while reducing damage to normal endometrium, thereby promoting endometrial repair and regeneration (Toropygin et al., 2011; Crosetti et al., 2019). Compared with traditional hysteroscopic surgery, laser surgery has the advantages of less bleeding and faster recovery, making it particularly suitable for handling weak or locally diseased areas of the endometrium, thereby improving endometrial thickness and function, which helps increase embryo implantation rates and pregnancy success rates (Kodaman and Arici, 2007; Papadopoulos and Magos, 2007). Ultrasound-guided surgical techniques monitor in real-time through ultrasound imaging, accurately locating the lesion site and assisting in the operation of surgical instruments, greatly enhancing the safety and effectiveness of the surgery. This technology can precisely excise lesions under minimally invasive conditions, avoiding damage to surrounding normal tissues while reducing the incidence of intraoperative complications. Ultrasound guidance is also widely used in auxiliary operations for endometrial regeneration treatment, such as cell grafting or drug injection, helping to achieve targeted and effective treatment (Yokomizo et al., 2021). The application of this technology not only optimizes surgical plans but also provides technical support for personalized treatment (He et al., 2012). Furthermore, with the development of biomedical engineering, endometrial organoids (EOs) as an emerging three-dimensional *in vitro* model provide a powerful tool for studying the pathological mechanisms and treatment strategies of thin endometrium. EOs can simulate the cellular composition and functional characteristics of human endometrium, helping scientists gain a deeper understanding of the regeneration and repair processes of the endometrium. By combining with laser and ultrasound-guided surgery, EOs are not only used for preoperative assessment and surgical plan design but also show great potential in monitoring endometrial function recovery and regeneration treatment after surgery (Jabri et al., 2025). Despite the diversity of existing treatment methods, there are still significant limitations: drug treatment has high resistance, with 35% of patients unresponsive (Park et al., 2021), and the recurrence rate of surgical intervention reaches 40% (Li et al., 2022). Biomaterials, due to their tunable physicochemical properties and biocompatibility, are expected to break through bottlenecks through mechanisms such as constructing biomimetic microenvironments and targeted delivery of growth factors (Rodríguez-Eguren et al., 2024).

### 3.3 Prospects for the application of biomaterials

Biomaterials have great potential for treating thin endometrium. Research indicates that biomaterials such as autologous adipose-derived mesenchymal stem cells and decellularized scaffolds have good effects in endometrial regeneration. These materials not only provide biocompatibility and structural support but also promote endometrial healing and regeneration (Kahyaoglu et al., 2024). For example, studies utilizing decellularized scaffolds for endometrial reconstruction have shown that this method can effectively improve endometrial thickness and function, providing a novel treatment strategy (Peng et al., 2024). Therefore, the application of biomaterials in the treatment of thin endometrium is expected to become a new treatment option (Table 1).

**TABLE 1:** Comparison of major treatment methods for thin endometrium.

Treatment type	Representative method	Mechanism of action	Advantages	Limitations	Clinical effect (pregnancy rate improvement)	References
Pharmacological Treatment	Estrogen (E2)	Directly promotes endometrial cell proliferation and gland development	Easy to use, low cost	Some patients respond poorly; long-term use may increase thrombotic risk	35%–50% (endometrial thickening ≥8 mm)	(Shi et al., 2023; Chen et al., 2024; Zhu et al., 2022)
	Granulocyte Colony-Stimulating Factor (G-CSF)	Activates stem cell differentiation, promotes angiogenesis	Local administration, few side effects	Requires intrauterine infusion; large individual variability in efficacy	40%–55%	(Kahyaoglu et al., 2024; Li X et al., 2020)
	Sildenafil	ncreases endometrial blood flow through NO pathway	Significant improvement in blood flow	Common side effects include headache and flushing	30%–45%	(Li X et al., 2020)
Surgical Treatment	Hysteroscopic Adhesiolysis	Mechanically separates adhesions, restores uterine cavity volume	Effective for adhesive thin endometrium	Possible recurrence post-surgery; risk of requiring second surgery	25%–40% (dependent on adhesion severity)	(Li et al., 2022)
	Autologous Endometrial Transplantation	Transplants healthy endometrial tissue to damaged areas	Long-term effects are stable	Donor site damage; high technical difficulty	50%–60% (small sample studies)	(Yokomizo et al., 2021)
Biological Treatment	Platelet-Rich Plasma (PRP)	Releases growth factors (VEGF, PDGF) to promote cell migration and angiogenesis	Autologous source, high safety	Preparation standards are not uniform; durability of effects is questionable	45%–65%	(Nazari et al., 2019; Yuan et al., 2024)
Biomaterials	Collagen Scaffold	Provides 3D structural support for cell attachment, sustained release of bioactive factors	Good biocompatibility, biodegradable	Low mechanical strength; needs to be combined with growth factors	60%–75% (combined with MSCs)	(Wang Y et al., 2023; Binlateh et al., 2022)
	PLGA Microspheres	Controlled release of drugs/cytokines, extends treatment window	Precise delivery, long-lasting	Degradation products may cause inflammation	50%–65% (experimental stage)	(Zhang L. et al., 2023; Xie et al., 2023)

## 4 Classification and characteristics of biomaterials

### 4.1 Natural biomaterials

Natural biomaterials refer to materials derived from living organisms, typically extracted from plants or microorganisms, which possess good biocompatibility, biodegradability, and low immunogenicity. These materials include collagen, hyaluronic acid, gelatin, fibrin, chitosan, and alginates, often containing cell recognition sites (such as RGD sequences) in their molecular structure, which can mediate cell-specific adhesion and activate intracellular signaling pathways. Therefore, they are widely used in the fields of tissue engineering and regenerative medicine. Collagen, as a major component of the extracellular matrix, not only provides structural support for cells but also promotes the proliferation and migration of endometrial epithelial cells and stromal cells through integrin-mediated signaling, significantly enhancing the regenerative capacity of the endometrium (Wang F. et al., 2023). Hyaluronic acid, with its excellent water retention and lubricating properties, can improve the microenvironment within the uterine cavity, providing a well-hydrated three-dimensional growth space for cells (Yin et al., 2023). In applications simulating endometrial stroma, researchers successfully constructed a three-dimensional artificial endometrial model using hyaluronic acid and collagen composite hydrogels, which exhibit good biomechanical properties and cell compatibility, promoting the directed differentiation of endometrial stem cells and the repair of endometrial tissue (Gao et al., 2021). Additionally, gelatin-based biomaterials, due to their ease of processing and good cell support capabilities, have also been used to prepare nanofiber scaffolds that promote the proliferation of endometrial stromal cells and angiogenesis, thereby improving the microenvironment of damaged endometrium (Huang et al., 2025). Relevant animal experiments and preclinical studies have shown that natural polymer materials can effectively promote the regeneration and functional recovery of the endometrium. For example, in rat and rabbit uterine injury models, the application of hyaluronic acid and collagen-based hydrogel carriers can significantly increase endometrial thickness, promote the generation of glands and blood vessels, reduce the degree of fibrosis, and significantly improve uterine structure and reproductive function (Huang et al., 2025; Zhang et al., 2020). This also indicates that natural polymer materials, as carriers for drugs or cells, can extend the retention time of therapeutic factors, improve targeted repair efficiency, and further enhance repair effects (Cai et al., 2022). Nevertheless, natural materials still have significant limitations: their sources are restricted (e.g., significant differences in collagen properties from different species or batches), they generally have poor mechanical strength, are easily degraded *in vivo*, and some xenogeneic materials may cause immune rejection reactions (Tripathi et al., 2023). Therefore, researchers often enhance their stability and mechanical properties through cross-linking modifications and composite with other materials, and actively explore recombinant protein technology to address the issue of inconsistent sources (Rodríguez-Eguren et al., 2024).

### 4.2 Synthetic biomaterials

Synthetic biomaterials are high molecular polymers artificially prepared by chemical synthesis methods, characterized by clear chemical structures, tunable physical properties, and good reproducibility. They mainly include polylactic acid (PLA), polycaprolactone (PCL), polyvinyl alcohol (PVA), polylactic-co-glycolic acid (PLGA), and polyethylene glycol (PEG). These materials can precisely control their degradation rates, mechanical strength, and hydrophilicity through molecular design, thus adapting to the needs of different tissue regeneration scenarios. For example, PLGA, as an FDA-approved biodegradable material, degrades into lactic acid and glycolic acid, which can be excreted through metabolic pathways. It has been widely used to construct controlled-release drug microspheres and tissue engineering scaffolds, achieving precise spatiotemporal control over the release behavior of growth factors (Yuan et al., 2024). PEG, as a hydrophilic polymer, can improve the hydration and cell compatibility of materials, commonly used to prepare hydrogels or as modifiers for other polymer materials to enhance their biological functions (Yin et al., 2023). The application advantages of synthetic materials in drug delivery and cell scaffolds are significant. Their controllable degradation allows for the sustained and stable release of drugs or growth factors, avoiding the short-term effects and systemic side effects of traditional drug therapies (Cai et al., 2022). In addition, synthetic polymers are easy to process into various forms, such as nanoparticles, microspheres, films, and 3D scaffolds, to meet different therapeutic needs. By adjusting the chemical composition and physical structure of the materials, the microenvironment for cell adhesion, proliferation, and differentiation can be optimized, promoting membrane repair (An et al., 2025). However, synthetic materials often lack bioactive sites and have poor cell affinity, often requiring surface modifications (such as grafting peptides or glycosaminoglycans) to improve cell adhesion and differentiation. Furthermore, their degradation process may cause a local decrease in pH, triggering an aseptic inflammatory response that hinders tissue repair (Park et al., 2021). Current research focuses on developing functional modification strategies and smart responsive synthetic materials to further enhance their biocompatibility and tissue integration capabilities.

### 4.3 Composite biomaterials

Composite biomaterials are hybrid materials formed by physically or chemically combining natural and synthetic materials, aiming to integrate the biological functionality of natural materials with the excellent mechanical properties and processability of synthetic materials. These materials not only possess adjustable degradation properties and mechanical strengths closer to natural tissues but can also impart active biological regulatory functions by introducing active components (such as growth factors, adhesion peptides, etc.). For example, combining collagen with polylactic acid can retain collagen’s ability to promote cell adhesion and proliferation while utilizing PLA’s mechanical support to resist mechanical stress within the uterine cavity, providing a more stable regenerative microenvironment for the endometrium (Sanyal et al., 2023). Similarly, gelatin-PCL composite electrospun fiber scaffolds can guide cell directional migration and organized tissue regeneration by mimicking the fibrous structure of the extracellular matrix. The performance of composite materials can be precisely customized by adjusting component ratios, spatial distributions, and interfacial bonding methods (Insuasti-Cruz et al., 2022). However, their preparation processes are often complex, facing challenges in phase compatibility, structural uniformity, and large-scale production, and the complex composition may introduce uncertain *in vivo* immune responses and metabolic behaviors (Insuasti-Cruz et al., 2022). Therefore, future research should focus on developing standardized, controllable composite processes and comprehensively assessing their long-term safety using multi-omics evaluation systems to promote the clinical translation of such materials in regenerative medicine fields like thin endometrium repair (Table 2).

**TABLE 2:** Classification and characteristics of biomaterials for thin endometrium repair.

Material type	Representative materials	Advantages	Disadvantages
Natural Materials	Collagen, Hyaluronic Acid	Good biocompatibility	Low mechanical strength
Synthetic Materials	PLA, PLGA	Strong controllability	Degradation products may cause inflammation
Composite Materials	Collagen-PLGA	Superior comprehensive performance	Complex preparation processes

## 5 Application research of biomaterials in thin endometrium repair

### 5.1 Application of collagen-based biomaterials

In recent years, collagen-based biomaterials have received widespread attention due to their excellent biocompatibility and biodegradability. Collagen is the most abundant structural protein in animals, providing a good growth environment for cells, demonstrating good biocompatibility and tissue regeneration capabilities (Wang F. et al., 2023; Binlateh et al., 2022). Multiple preclinical studies have shown that collagen not only serves as an important component of the extracellular matrix, playing a scaffolding role in the restoration of the endometrial structure, but also promotes endometrial regeneration and functional recovery by binding with stem cells or other bioactive factors. Researchers utilized human umbilical cord mesenchymal stem cells (hUCMSCs) loaded collagen scaffolds transplanted into a rat model of intrauterine adhesions (IUA) caused by mechanical injury, and the results showed that this composite material significantly increased the thickness, gland number, and vascular richness of the damaged endometrium, while significantly reducing the degree of endometrial fibrosis. At the molecular level, hUCMSCs-loaded collagen scaffolds can upregulate the expression of vascular endothelial growth factor (VEGF), integrin β3, interleukin family member LIF, and insulin-like growth factor 1 (IGF-1), ultimately improving endometrial receptivity and effectively promoting endometrial regeneration and functional recovery (Gao et al., 2025). In addition, the hydrogel system formed by recombinant human type III collagen (RHC) and hyaluronic acid (HA) overcomes the defects of easy degradation and instability of type III collagen *in vivo*, promoting the proliferation and adhesion of endometrial cells through sustained release, significantly enhancing endometrial regeneration and recovery of fertility. This composite hydrogel also exhibits good anti-fibrotic effects, effectively preventing abnormal fibrosis during the endometrial healing process, thus providing a new strategy for the treatment of endometrial damage (Wei et al., 2024). For the full-thickness uterine injury model, the transplantation of collagen carriers combined with endometrial perivascular stem cells (En-PSCs) and the active component hydroxysafflor yellow A (HSYA) can significantly promote endometrial repair. Specifically, this is manifested as reduced fibrosis, increased endometrial thickness, regeneration of the myometrium, promotion of angiogenesis, and ultimately improved pregnancy rates. Mechanistic studies show that this combination promotes angiogenesis and endometrial repair by activating the NRG1/ErbB4 signaling pathway, providing a molecular basis for the application of collagen-based materials combined with cell therapy (Li et al., 2024).

### 5.2 Research progress of polylactic acid (PLA) and its derivatives

Polylactic acid (PLA) and its derivatives, as a class of biodegradable and low immunogenic materials, have been widely used in the field of biomedical science in recent years due to their excellent biocompatibility and tunable mechanical properties, including drug delivery, tissue engineering scaffolds, and regenerative medicine (Chen et al., 2023; Li G. et al., 2020; Akhila et al., 2025). PLA can be synthesized and modified through various methods to prepare various structural forms, such as nanofibers, 3D printed scaffolds, and films, to meet different clinical needs (Li G. et al., 2020; Ekinci et al., 2021). At the same time, the mechanical properties, thermal stability, and bioactivity of PLA materials can be further optimized through nanocomposite technology or by compounding with other bioactive substances, enhancing their application performance in the biomedical field (Azarian et al., 2025; Binaymotlagh et al., 2025). In the treatment of thin endometrium, PLA and its copolymers are applied to construct porous scaffolds and microsphere carriers, providing a three-dimensional growth environment for endometrial cells and achieving sustained release of drugs or growth factors, thereby promoting the regeneration and recovery of function of endometrial tissue (Zhang et al., 2023; Xie et al., 2023; Wang J. et al., 2024). Studies have found that scaffolds constructed using PLA materials combined with human umbilical cord mesenchymal stem cells and collagen hydrogel can extend the retention time of cells in the uterus, significantly improving endometrial thickness and fertility (Wang J. et al., 2024). Hormonal drugs (such as 17β-estradiol) can be loaded into PLGA microspheres and integrated into bioactive scaffolds, achieving sustained drug release that matches the female menstrual cycle, promoting the proliferation and regeneration of endometrial cells (Chen et al., 2020). The anti-inflammatory drug Pentoxifylline (PTXF) loaded in PLGA has been shown to effectively improve the thin endometrium in model animals, promoting the recovery of endometrial and myometrial thickness, demonstrating more significant tissue repair effects than traditional medications (Saleem Raheem et al., 2023). In addition, PLA composites optimize the spatial support and bioactive release for endometrial repair by regulating the degradation rate and mechanical properties, reflecting potential advantages in the repair of thin endometrium (Shuai et al., 2023; Mahajan et al., 2024). These studies indicate that PLA and its derivatives have great application potential in the repair of thin endometrium and are expected to become a new option for clinical treatment in the future.

### 5.3 Exploration of other novel biomaterials

In addition to collagen and polylactic acid, research on other novel biomaterials in the repair of thin endometrium is also continuously deepening. New materials such as bioactive glass, polyurethane, and alginates are gradually being applied in endometrial repair research due to their excellent biocompatibility and ability to promote cell proliferation. These materials can not only provide good scaffold structures but also promote endometrial regeneration by modulating the microenvironment. Research by Song S et al. has found that alginate-based biomaterials can effectively promote the proliferation and migration of endometrial cells, and their positive effects on endometrial repair have been verified in animal models (Song et al., 2024a; Song et al., 2024b). Furthermore, the functional design of biomaterials has also become a research hotspot, further enhancing their effectiveness in clinical applications by combining growth factors, drug carriers, and other functions. In summary, the exploration of other novel biomaterials provides more possibilities for the repair of thin endometrium.

## 6 Mechanisms of biomaterials in thin endometrium repair

### 6.1 Promoting cell proliferation and migration

Biomaterials really help endometrial cells grow and move by simulating the physicochemical properties of natural extracellular matrix (ECM), providing suitable microenvironments (Ge et al., 2015; Salehi Abar et al., 2024; Qiao et al., 2024). In the repair process of thin endometrium, biomaterial scaffolds not only provide physical support for endometrial epithelial and stromal cells but also activate downstream signaling pathways such as FAK/PI3K/Akt by binding to cell surface integrin receptors through their inherent bioactive ligands (e.g., RGD peptides), thereby regulating the expression of cell cycle proteins (e.g., Cyclin D1) and driving cells from the G1 phase into the S phase, accelerating cell proliferation (Lanza et al., 1997; Baldin et al., 1993; Qiao et al., 2022). Studies have shown that collagen-based materials, rich in natural ECM components, can significantly enhance the proliferation rate of endometrial stromal cells (up to 1.5–2.0 times) through α2β1 integrin-mediated signaling and enhance cell migration ability by regulating the secretion of MMP-2/MMP-9, thereby accelerating the re-epithelialization process of the damaged endometrium (Yang et al., 2024). Additionally, some functionalized biomaterials (e.g., grafted EGF or VEGF-mimicking peptides) can further strengthen the proliferative/migratory effects through sustained activation of growth factor receptors.

### 6.2 Improving blood supply and nutritional support

Biomaterials improve local blood supply through various mechanisms during the repair process, providing necessary nutritional and oxygen support for endometrial regeneration. On one hand, biomaterials can serve as controlled-release carriers for angiogenic factors, continuously releasing VEGF, FGF-2, and Angiopoietin-1 to activate the VEGFR2/FGFR signaling pathways on endothelial cells, promoting endothelial cell proliferation, migration, and lumen formation (Modaresifar et al., 2018; Wernike et al., 2010; Li et al., 2015). On the other hand, the three-dimensional porous structure of the materials provides physical space for the migration of vascular endothelial cells and the establishment of a new vascular network (Xiao et al., 2015). Research has found that PLA-based porous scaffolds can continuously release lactic acid metabolic products by regulating the degradation rate, inducing upregulation of HIF-1α expression, and subsequently promoting VEGF secretion and angiogenesis (Rodríguez-Eguren et al., 2024). This newly formed vascular network not only improves the perfusion and oxygenation status of the repair area (increasing local oxygen partial pressure by 30%–50%) but also ensures the timely removal of metabolic waste, creating favorable conditions for the metabolic activities and functional recovery of endometrial cells (Grapensparr et al., 2015).

### 6.3 Regulating local microenvironment

Biomaterials actively regulate the local microenvironment of the damaged area through their physicochemical properties and bioactive components, influencing cell behavior and tissue repair outcomes (Amani et al., 2024; Zhu et al., 2021; Bian et al., 2023). In terms of immune regulation, biomaterials can modulate the recruitment and polarization state of immune cells through physical and chemical properties such as surface topology, hydrophilicity/hydrophobicity, and ionic charge (Li et al., 2021; Asadi Tokmedash et al., 2025; Hotchkiss et al., 2016). For example, gelatin-based hydrogels can promote macrophage polarization towards a reparative M2 phenotype through TLR2/4 signaling pathways, increasing the secretion of anti-inflammatory factors such as IL-10 and TGF-β while reducing levels of pro-inflammatory factors such as TNF-α and IL-1β, thereby alleviating inflammatory responses and promoting the formation of a reparative microenvironment. In terms of extracellular matrix remodeling, biomaterials can inhibit excessive collagen deposition and fibrosis by balancing the activity of MMPs/TIMPs, promoting the regeneration of normal ECM (Jiang et al., 2021; Yang et al., 2018; Taskin et al., 2021). Additionally, bioactive molecules released from the materials (such as SDF-1) can recruit endogenous stem cells to the injury site and promote their differentiation into endometrial cells through pathways such as Wnt/β-catenin, fundamentally restoring the regenerative capacity of the endometrium (Liu et al., 2024).

## 7 Challenges and prospects in clinical applications

### 7.1 Barriers to clinical translation of biomaterials

There are several hurdles to getting biomaterials into clinical use, primarily including material selection, production processes, and strict requirements for biocompatibility. Many laboratory-grade materials used in studies are not suitable for direct application in humans, leading to delays in clinical translation. For example, biomaterials commonly used in neurosurgery, such as Duragen Plus™, although performing well in laboratory settings, still require further validation of their safety and efficacy in clinical applications (Finch et al., 2020). Additionally, the interaction between biomaterials and human tissues may trigger immune responses, affecting the functionality of the materials and the safety of patients (Angeletti et al., 2019). Therefore, developing materials with good biocompatibility and functionality is key to achieving clinical translation. At the same time, the complex approval processes for biomaterials by regulatory agencies increase the difficulty of clinical applications, requiring researchers to fully consider these factors during the design and testing phases to enhance the clinical application potential of the materials (Xu et al., 2024).

### 7.2 Assessment of safety and efficacy

In the clinical application of biomaterials, it's super important to assess safety and effectiveness. Although many biomaterials show good performance in in vitro experiments, their *in vivo* performance may vary significantly. The design of clinical trials must strictly adhere to scientific standards to ensure the reliability and reproducibility of the data (Peng et al., 2021). For instance, biomaterials used in immunotherapy need to assess their *in vivo* biodegradability, toxicity, and impact on the immune system (Xue et al., 2022). Therefore, the long-term safety of biomaterials also needs to be evaluated through long-term follow-up studies to timely identify potential side effects and adverse reactions (Blanc et al., 2020). Efficacy assessment should not only focus on treatment effects but also comprehensively consider patients’ quality of life and the acceptability of treatments, providing a more comprehensive perspective for the clinical application of biomaterials (Sabra et al., 2021).

## 8 Conclusion

In summary, biomaterials can effectively promote the regeneration and repair of the endometrium, improving patients’ fertility. This finding brings hope to women facing difficulties in pregnancy. Therefore, the introduction of biomaterials is undoubtedly an important breakthrough in the field of modern obstetrics and gynecology. However, it should be noted that there are certain discrepancies in the results between different studies. These discrepancies may arise from multiple factors, including material selection, differences in repair methods, and individual differences among patients. Therefore, to better understand the mechanisms of action of biomaterials and their efficacy in clinical applications, promoting the integration of biomaterial technology with clinical needs will be an important direction for future research.
