# Correction: Advances in using biomaterials for repairing thin endometrium

**DOI:** 10.3389/fbioe.2025.1749049

**Published:** 2025-12-05

**Authors:** Huizhen Li, Feihong Hu, Fuchen Xie, Xuedong Chen, Honglian Wu

**Affiliations:** Reproductive Medicine Center, Lishui Hospital of Wenzhou Medical University, The First Affiliated Hospital of Lishui University, Lishui People’s Hospital, Lishui, Zhejiang, China

**Keywords:** biomaterials, thin endometrium, tissue engineering, regenerative medicine, repair

Author Feihong Hu was erroneously assigned as **Corresponding Author**. The correct corresponding author is Honglian Wu.

The author Feihong Hu was erroneously omitted as **Equal Contributing** author and co-first author. Authors Huizhen Li and Feihong Hu equally contributed to this manuscript as the first authors.

The original article has been updated.

